# SOLACE: A Psychosocial Stigma Protection Intervention to Improve the Mental Health of Parents of Autistic Children—A Feasibility Randomised Controlled Trial

**DOI:** 10.1007/s10803-020-04498-0

**Published:** 2020-04-22

**Authors:** Annemarie Lodder, Chris Papadopoulos, Gurch Randhawa

**Affiliations:** Institute for Health Research, University of Bedfordshire, Putteridge Bury, Hitchin Road, Luton, LU2 8LE UK

**Keywords:** Autism, Feasibility, Stigma, Parents/carers, Mental health, RCT, Intervention

## Abstract

This study presents findings from a feasibility trial, testing an 8-week psychosocial stigma protection intervention (SOLACE) designed to improve the mental health of parents of autistic children. Seventeen parents were stratified then randomly assigned to either SOLACE (n = 9) or control group (n = 8). Retention and adherence rates were excellent with minimal missing data suggesting SOLACE had good acceptability and feasibility. Quantitative analysis revealed that mental health scores had significantly improved for those who took part in SOLACE compared to no significant changes for control group participants. In addition, changes in secondary outcome measures (e.g. stigma, self-esteem and self-compassion) were in favour of SOLACE. Focus group interviews revealed that SOLACE was acceptable to parents. Results suggest that a full randomised controlled trial is warranted.

## Introduction

Caring for an autistic child can be challenging for parents (e.g. Divan et al. 2012; Kinnear et al. 2016; Ntswane and VanRhyn 2007; Schall 2000), with parents reporting poorer mental health than the general population (Baker et al. 2011; Benson and Karlof 2009; Griffith et al. 2010). A meta-analysis of twelve studies conducted by Hayes and Watson (2012) demonstrates how parents of autistic children experience more parenting stress than those of children with other disabilities with a mean effect size of 0.64 (95% CI 0.25–1.03).

Factors that have been reported to contribute to this include a child’s level of cognitive impairment, externalising behavioural problems (e.g. aggression, meltdowns) and internalised distress, hyperactivity, eating difficulties, toileting, strict routines, and social difficulties (Brown et al. 2011; Gray 1994; Hall and Graff 2011; Ingersoll and Hambrick 2011; Koegel et al. 1992; Lecavalier et al. 2006; Lee et al. 2008; Little and Clark 2006; Tomanik et al. 2004). Additionally, research suggests that the stigma surrounding autism and the way that the public reacts to their child is particularly stressful for parents (e.g. Crabtree 2007; Green 2007; Gray 2002) and has negative consequences for their well-being (Papadopoulos et al. 2018). Studies have shown that up to 95% of parents report having experienced stigma (Kinnear et al. 2016) and recently, the impact of stigma upon parental well-being is receiving increased recognition.

Stigma is a social phenomenon where certain groups (such as autistic people) are marginalised and devalued because their characteristics, practices, or values differ from the dominant cultural group (Ali et al. 2012). Stigma extends to those closely affiliated with a stigmatised individual and is defined as courtesy stigma (Goffman 1963) or family stigma (Moses 2014) and manifests itself in parental blame (Dababnah and Parish 2013; Mak and Kwok 2010; Ryan 2008) social avoidance (Moses 2014) and rejection (Kinnear et al. 2016).

A relatively new concept in the autism stigma literature is the self-stigma, or internalisation of courtesy stigma parents experience, which has been defined as ‘affiliate stigma’ (Mak and Cheung 2008). Self-stigma and its detrimental consequences for mental health have received much recognition in the fields of serious mental illness (e.g. Corrigan et al. 2009; Lucksted et al. 2011; Yanos et al. 2015). According to Corrigan and Watson (2002)’s 3A model, the self-stigma process starts with the awareness of stereotypes which are then subsequently agreed with and applied to oneself. Mitter et al. (2019) propose that the internalisation of stigma in parents of autistic children follows a similar path. Research looking at affiliate stigma in parents of autistic children found that affiliate stigma positively relates to psychological stress and burden (Mak and Kwok 2010; Chiu et al. 2013). Further, affiliate stigma among parents of autistic children is higher than that of any other disability (Ali et al. 2012; Mak and Kwok 2010; Werner and Shulman, 2015).

A recent systematic review from Papadopoulos et al. (2018) produced compelling evidence pertaining to the negative relationship between stigma and mental health among parents and carers of autistic children. Papadopoulos et al. (2018) reviewed five qualitative and seven quantitative studies representing 1442 parents of carers across a wide range of geographical settings and found stigma to be directly and consistently related to depression, anxiety, psychological distress and general mental health. Further the literature suggests several modifiable psycho-social variables that moderate the stigma-mental health relationship, namely self-esteem (Cantwell et al. 2015; Mak and Kwok 2010; Werner and Shulman 2013), social support (Ali et al. 2012; Broady et al. 2017; Cantwell et al. 2016; Mak and Kwok 2010; Werner and Shulman 2013), self-blame (Mak and Cheung 2008; Mak and Kwok 2010; Werner and Shulman 2013, 2015); positive meaning in caregiving (Broady et al. 2017; Werner and Shulman 2013) and self-compassion (Chan and Lam 2017; Wong et al. 2016; Zhou et al. 2018). Research suggests that cultivating self-compassion may be particularly beneficial for this population (Beer et al. 2013; Neff and Dahm 2015; Neff and Faso 2014). Wong et al. (2016) note that self-compassion can serve as an internal coping resource without having the family carer rely on external sources of help. Self-compassion has been described as an emotional regulation strategy that may allow parents to acknowledge and understand negative emotional reactions implicated in affiliate stigma (Wong et al. 2016). Wong et al (2016) argue that self- compassion helps parents deal with daily stressors including stigma.

Thus, given the evidence that highlights stigma as a key risk factor of poor parental mental health and wellbeing (Papadopoulos et al. 2018), increasing parents and carers’ resilience and protection to stigma should in theory have a positive impact on their mental health and subsequently their caregiving abilities. Furthermore, early interventions focused on new carers could be particularly important since they may be more vulnerable to self-blame during the early stages of diagnosis and, as such, to self-stigma (Lodder et al. 2018). New carers are more vulnerable to the misconceptions associated with autism given this may be the first time they have ever encountered autism (Papadopoulos et al. 2018). Therefore, they may be more prone to misconceptions, myths, and negative stereotypes.

To our knowledge, there are currently no interventions available that have been designed to support parents of autistic children cope with the stigma they experience and/or prevent the internalisation of stigma. To address this, a blended face-to-face and online psychosocial ‘stigma protection’ intervention, that aims to improve the mental health of parents and carers of autistic children through strategies designed to help parents/carers resist stigma, was developed (‘SOLACE’). SOLACE was developed following the Medical Research Council guidelines for the development of complex intervention (Craig et al. 2008). To encourage input from the autism community in the design of the intervention, an online survey was carried out to explore their views and suggestions to make an intervention more successful (Lodder et al. 2019). Respondents suggested that parents’ would benefit from ‘ready-made’ phrases or information available to react to instances of stigma from the public, other family members, and professionals which was incorporated into the intervention. (Lodder et al. 2019).

A blended format was adopted because parents and carers of autistic children are known to experience barriers for attending face to face groups such as restricted time availability, travel and childcare issues (Clifford and Minnes 2013; Lodder et al. 2019; Whitebird et al. 2011). Further, existing evidence indicates that a blended format intervention can be both practical and effective for this population (Hall and Bierman 2015; Lipman et al. 2011; Lodder et al. 2019; Clifford and Minnes 2013).

Because no comparable interventions were available it was unknown what will be effective, acceptable and achievable with regard to the logistics and practicalities of such an intervention. Therefore, it was necessary to carry out a feasibility study to assess the feasibility and acceptability of the designed intervention. The primary aim of the current study was to evaluate the feasibility and acceptability of SOLACE. Secondary aims were to explore the preliminary impact of the intervention on the mental health of the parents and carers as well as other outcome measures including courtesy and affiliate stigma; self-esteem; self-compassion, positive meaning of caregiving; self-blame; social support; and social isolation.

## Methods

### Trial Design

A mixed methods design was employed to evaluate the feasibility, acceptability and preliminary impact of the intervention. A parallel randomised controlled trial (RCT) that compared changes in outcomes of interest among parents allocated to the SOLACE experimental group against parents or carers allocated to the control group (no intervention) was carried out. After informed consent was obtained, all eligible participants received a baseline questionnaire. Participants were stratified based on gender and frequency of use of support groups (ranging from 0 (never) to 4 (often)) to minimise imbalance between the two groups. Randomisation was conducted by an independent researcher via computer generated allocation. Double-blinding of group allocation was not practically possible since the intervention was facilitated by the first author. Measures were obtained immediately post intervention and six weeks post intervention. Participants allocated to the control group received an information pack containing the materials that were discussed during the intervention after the final data collection.

The Consolidated Standard of Reporting Trials (CONSORT) extension to pilot trials was followed for reporting the trial (Schulz et al. 2010).The trial was registered at the ISRCTN Registry (www.isrctn.com) with registry number ISRCTN61093625.

### Ethics

Ethical approval was granted by the University of Bedfordshire’s Institute for Health Research Ethics Committee (ref: IHREC812) and the study complied with Europe’s General Data Protection Regulation and Data Protection Act 2018 (DPA 2018). Participants were told that in the event that they do feel distress that they can withdraw from the study at any time. In such cases participants would be offered the opportunity to debrief with the facilitator, as well as a list of mental health resources (including services across the county and relevant online support groups). To minimise the potential negative emotional impact for the facilitator, a reflective diary was kept and sessions were debriefed with the research team.

### Participants

This study targeted parents and carers of children up to the age of 10 years who had been diagnosed as autistic within the past 12 months or were still undergoing diagnostic assessment at the time of recruitment. This age range of children was chosen given the intervention’s focus on new carers and early intervention with this population. The target sample size for this feasibility trial was 24. There are no specific guidelines for sample sizes of feasibility studies or definitive guidelines for group sizes of psychosocial group interventions although common practice is for group sizes to range between 6 and 12 participants (e.g. Hayes and Watson 2012; Frantz et al. 2018; Lucksted et al. 2011).

Participants had to be able to travel to Bedfordshire for the face to face meetings and have access to a device that could enable them to take part in the online meetings (e.g. mobile, tablet, PC with a camera and microphone). Participants also had to be able to speak and understand English. The recruitment period ran from 15th June 2018 until 30th September 2018 and took place through a number of venues including special needs centres and local parenting groups in Bedfordshire, community forums on social media such as autism and special needs Facebook support groups, and advertisement via Autistica’s autism research registry and snowballing techniques.

### The Intervention: SOLACE

The intervention was developed in line with the Medical Research Council’s guidelines for developing complex interventions (Craig et al. 2008). It was hypothesised that a multi-component intervention using psycho-education, cognitive restructuring strategies and compassion focused techniques should see protection against internalising stigma and reduce the harmful effects of stigma and subsequently improve parental mental health. The intervention incorporates a variety of techniques including lecture, group discussions, guided activities and sharing of experiences. In line with Corrigan and Watson (2002) 3A model, helping parents identifying ways to discount the stigma they experience as holding no truth or value will be beneficial in reducing self-stigma. The group format as well as the learning about the importance of social support should enhance feeling of belonging and increase perceived social support which should further protect against the harmful impact of stigma. Further, cultivating self-compassion should reduce self-blame and self-stigma in parents. An example of an easy to implement strategy is the ‘how would you treat a friend’ exercise. Parents are encouraged to apply the same kindness or compassion they would towards others, to themselves. Based on the input from the autism community, parents were also encouraged to discuss ways to respond to stigmatising situations. The intervention included video material of parents of autistic children talking about ways of coping developed by Sesame Street’ titled ‘See Amazing in All Children’ (https://autism.sesamestreet.org/). This material was developed to reduce stigma and build positive perceptions about autism and designed with help from the autism community (See Amazing, executive summary 2017). The use of video material especially designed for parents of autistic children has been shown effective help reduce autism stigma among the public but also among parents of autistic children (See Amazing, executive summary 2017).

Eight weekly sessions were delivered either face to face (session 1, 4 and 8) or via videoconference (session 2, 3, 5, 6 and 7) to limit barriers to attendance and increase retention rates. The sessions were each based around a theme (see Table 1) followed by free sharing time during which parents were could discuss topics of their choice or ask the group for information or advice. An overview of SOLACE’s session structure, focus and content is shown in Table 1.

**Table 1:** Summary of intervention content and aims

#	Topic	Theme	Aim
1	Introduction	Exploration of autism and autism stigma. Autism myths and stereotypes will be challenged through psycho-education and a group discussion about common stereotypes	To make introductions and provide an overview of the sessions To increase knowledge about autism To reduce feelings of self-blame and increase self-esteem
2	Coping with autism stigma	Group discussions of experiences of stigma using quotes from other parents. “How would you treat a friend” exercise	To develop skills how to recognise and cope with stigma and to prevent internalising stigma
3	Positive meaning of caregiving	Video clips of parents of autistic children showing how having an autistic child has changed them. Encourage group reflection	To increase the positive meaning associated with the caregiving role and increase self-compassion
4	Resilience and self-esteem	Group task to work together to find “key phrases and responses” to stigmatising situations	To increase resilience and increase self-esteem. To reduce social isolation and increase feeling of belonging
5	Social support	Stress the importance of social support and discuss together how social support could be utilised and or improved	To stress the importance of social support to help reduce social isolation
6	Compassion and acceptance	Discuss the importance of self-care and self-compassion as well as acceptance. Use video clips of how other parents have achieved this	To increase feelings of self-compassion and acceptance
7	Coping with autism stigma part 2	Discuss the automatic thought cycle (self-fulfilling prophecy). Group discussion on how we can break this cycle	To further develop skills how to recognise and cope with stigma and prevent internalisation of stigma
8	Next steps	Group discussions on how to disclose the diagnosis to others and to provide list of support for future	To further increase self-esteem and reduce social isolation To conclude the sessions and provide “next steps” for future reference

During the intervention period, a ‘secret’ Facebook group, which only the experimental group participants have access to and cannot be identified by others through searching, was used to support the intervention and to boost retention rates. After each session, information relevant to the session was shared in the Facebook group and participants were encouraged to use the group to ask each other questions, or to share their experiences or concerns.

A week before the start of the intervention, participants were sent detailed information about timings and locations, instructions on how to join the ‘secret’ Facebook group and how to use the videoconferencing program ‘Zoom’ (www.zoom.us). The intervention ran from 2nd October 2018 to 20th November 2018. The sessions were facilitated by a PhD student (##) who was experienced working with families of autistic children. During the face to face sessions, an assistant facilitator (a Health Psychology Masters student), was present to take notes and assess implementation fidelity.

### Measures

#### Feasibility

Feasibility was examined through recruitment rates, willingness to be randomised, retention and attendance rates, and missing data. We aimed to recruit 24 participants that were willing to be randomised and, based on previous intervention studies with this population (Hall and Bierman 2015) we viewed the trial design as feasible if all of the following thresholds were exceeded: ≥ 60% retention rates; average SOLACE attendance rates of ≥ 50% and each outcome measure having less than 50% missing data. Adverse events and fidelity to the implementation manual were measured by checklists developed for the study which were completed by the facilitators and checked by the wider research team.

#### Acceptability

Acceptability was assessed through a qualitative focus group interview and defined as acceptable if the majority of feedback was positive. A qualitative focus group interview was carried out covering the structure and formant, intervention content, future suggestions and outcome measures. The focus group was led by the SOLACE facilitator. The questions asked included, “What did you think about the mode of delivery of SOLACE” and “Do you think SOLACE was beneficial to you?” Seven participants took part in the focus group interview and one participant was interviewed individually at a later date due to holiday arrangements. The majority of participants (n = 7) had indicated that they preferred for the focus group to be online and thus, to maximise attendance, both the focus group and individual interview were conducted online via Zoom videoconference software. The focus group and interview lasted approximately 60 min and 30 min respectively. Framework analysis (Ritchie and Spencer 1994) was used to infer main themes from the transcripts. Framework analysis begins deductively from the objectives set for the focus groups, but also uses an inductive approach from the accounts of the participants, i.e. new themes can emerge from the discussion with the parents. All participants were given pseudonyms to protect their identity.

### Effectiveness Outcomes

The outcome measures used to assess the preliminary impact of SOLACE are presented below and were collected at baseline, post intervention and at six week follow up.

*Mental Health* was measured with the validated and reliable 5-item ‘Mental Health Inventory’(MHI-5) (Berwick et al. 1991) which is the ‘mental health’ subscales of the widely used Medical Outcomes Study 36-Item Short Form Health Survey (SF-36). Participants were asked to rate on a six-point scale how often they felt as described during the past month e.g. ‘How much of the time, during the past month, have you felt so down in the dumps that nothing could cheer you up?’ Scores ranged from 1 (all of the time) to 6 (none of the time) and an average score was calculated to compute a total MHI transformed score ranging from 0–100 (MHI Total score = [(mean MHI-1)*100]/5) with lower scores indicating poorer mental health. The internal consistency in the current study was good (α = 0.93).

*Courtesy Stigma* Levels of experienced courtesy stigma were measured using the Perceived Courtesy Stigma Scale (PCSS) (Chan and Lam 2017) which contains seven items adapted from the Devaluation of Consumer Families Scale (Struening et al. 2001). Parents rate each item on a four-point Likert scale ranging from 0 (strongly disagree) to 3 (strongly agree). A mean score was computed, with higher scores suggesting higher levels of perceived courtesy stigma. Items include statements like: ‘Most people blame parents for their child being autistic’. The PCSS has been used with parents of autistic children and demonstrated high validity and reliability (Chan and Lam 2017). For the current study the reliability score was equally good (α = 0.88).

*Affiliate Stigma* Affiliate Stigma was measured using an adapted version of the Affiliate Stigma Scale devised by Mak and Cheung (2008). This original 22-item Chinese scale was adapted to be suitable for UK parents and carers. The phrase ‘family member with mental illness/intellectual disability’ was replaced with ‘autistic child’. Three items with the lowest factor loadings were dropped in order to decrease the number of negatively worded items based on work by Werner and Shulman (2015). An example item is, ‘The behaviour of my child makes me feel embarrassed’. Each item is rated on a 4-point Likert scale ranging from 1 (strongly disagree) to 4 (strongly agree). A mean score was calculated with higher scores indicating higher levels of affiliate stigma. Internal consistency for the current study was high (α = 0.90).

*Self-esteem* Rosenberg’s 10-item Self-Esteem Scale (Rosenberg 1979) was used to measure self-esteem. Participants were asked to rate how 10 statements applied to them on a scale from 0 (strongly disagree) to 3 (strongly agree). An example statement is, ‘I feel that I have a number of good qualities’. An overall index can be calculated by adding scores. Scores can range from 0–30 with score below 15 are considered as low self-esteem. The internal consistency was high in the current study (α = 0.91).

*Self-Compassion* Self-Compassion was measured with the Self-Compassion Scale–Short Form (Raes 2011) modified version of Self-Compassion Scale (Neff 2003). The 12 items are rated on a 5-point response scale ranging from 1 (almost never) to 5 (almost always). An average score was calculated to provide a mean self-compassion score. The SCS-SF has demonstrated good internal consistency (α > 0.86) and a near-perfect correlation with the long form SCS (r ≥ 0.97). The internal consistency in the current study was good, α = 0.86.

*Positive Meaning of Caregiving* Based on Werner and Shulman’s (2015) study, positive meaning in caregiving was measured with 11 items taken from parents’ qualitative responses to the question, ‘How has caregiving for your child affected your life?’ in a study by Meyers et al. (2009) and also from the 5-item Perceived Benefits scale constructed by Green (2007). A sample item is, ‘Being a parent/carer to an autistic child has taught me kindness, patience and happiness.’ Each item was rated on a 5-point scale ranging from 0 (strongly disagree) to 4 (strongly agree) and an overall index of the mean score was computed. The internal consistency for this scale was high both for the current study (α = 0.91) and in Werner and Shulman’s study (α = 0.86).

*Self-blame* A shortened version of the Self-blame and Responsibility Scale developed by Mak and Kwok (2008) was used. The scale was translated from Chinese and slightly adapted to make it more suitable for the UK population. For example, the item, “I blame myself for allowing my child to have autism-related negative behaviour” was replaced with “Whenever my child shows autism-related behavioural problems, I would blame myself”. Ratings were made from 1 (strongly disagree) to 7 (strongly agree) and the mean score was calculated, with higher scores indicating higher rates of feelings of self-blame. The internal consistency was good (α = 0.75).

*Social Support* To measure perceived social support the Medical Outcomes Study: Social Support Survey (Sherbourne and Stewart 1991) was used. This scale measures four components of perceived availability of social support, including (1) Emotional support/Informational support, (2) Tangible support (3) Positive social interaction, and (4) Affectionate support. A sample item is, ‘Someone to prepare your meals if you were unable to do it yourself’ and scores ranged from 1 (none of the time) to 5 (all of the time). Scores were added to compute a total score with higher scores indicating higher perceived social support. It has shown good reliability (α = 0.85) and validity on total scale (α = 0.88) as well as subscales in previous studies with caregivers (Sherbourne and Stewart 1991) and excellent internal consistency score in the current study (α = 0.95) too.

*(Subjective) Social Isolation* The Short-form UCLA Loneliness Scale (Russell et al. 1980) was used to measure social isolation. The scale consists of 8 items with scores ranging from 1 (never) to 4 (often). A total score was calculated with higher scores indicating higher feelings of isolation. An example item is “How often do you feel that there is no one you can turn to?” The internal consistency in the current study was high with a Cronbach alpha of 0.86.

*Other Baseline Measures* Data on general demographic variables such as age, gender, ethnicity, marital status, income, religious affirmation, educational level, child’s primary diagnosis, how many children, age of child, and current use of (online) support groups was collected.

### Statistical Analysis

IBM SPSS v23 (IBM Corporation, 2015) software was used to analyse all quantitative data. Descriptive statistics were used to describe rates and patterns of recruitment, follow-up and retention rates, and willingness of randomisation. Descriptive statistics were also used to describe the sample. Independent *t*-tests at group level for continuous data and Pearson’s χ^2^ tests for categorical data were used to compare demographic and outcome measure differences between groups at baseline.

As suggested by the CONSORT guidelines, an intention to treat (ITT) analysis was carried out*,* subject to the availability of data. When there was no data for outcome measures or more than half of an outcome measure or domain was missing, it was classed as missing data and the outcome measure or domain was removed from the final ITT analysis. When more than half of the values on a single domain were recorded, sample mean imputation was used.

Sharpiro-Wilks was used to confirm the assumption of normality (Ghasemi and Zahedias 2012). All quantitative outcome measures met the assumptions of normality apart from ‘social isolation’ which was slightly negatively skewed. Because this is a well validated outcome measure and the remaining outcome measures did meet the normality assumptions, it was decided to transform the social isolation scores to be able to conduct parametric tests on all outcome measures.

A one-way repeated measures analysis of variance (ANOVA) was used to compare the mean values of the outcome variables over the three time points within both groups. Based on recommendations for studies of an exploratory nature, no Bonferroni adjustments for type 1 error were applied (Althouse 2016; Armstrong 2014; Perneger 1998). Where significant, repeated t-tests were carried out to explore the changes in outcome compared to baseline measures. Between group differences were also analysed at T1 and T2 using independent t-tests.

## Results

### Participation and Attrition

**Fig. 1:**
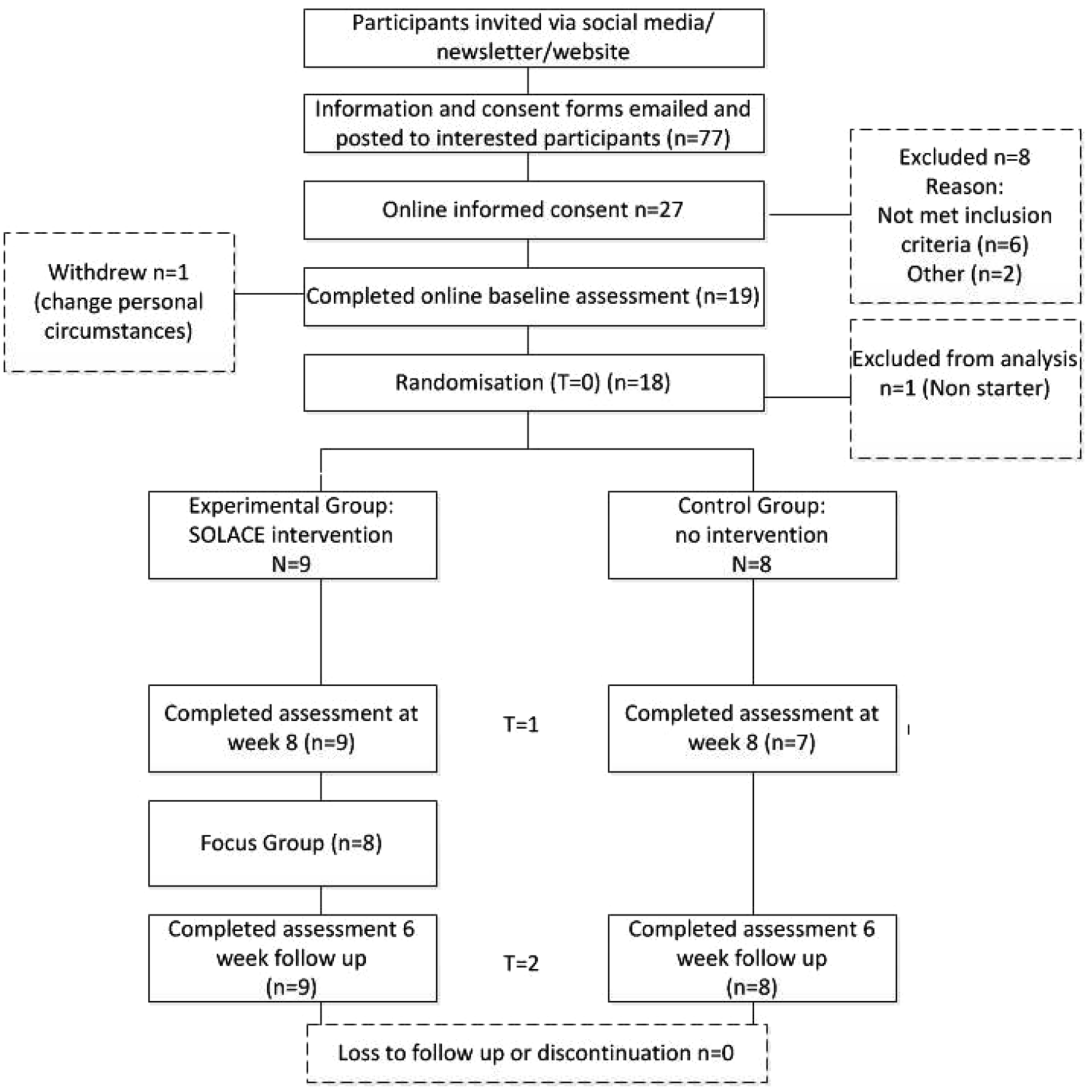
CONSORT participant flow chart

Seventy-seven people accessed the online information, of which 27 subsequently provided informed consent. Six people failed to meet the eligibility criteria due to their child being aged 10 years or over (n = 3) or because the diagnosis was obtained more than 12 months ago (n = 3) leaving 21 eligible participants. Nineteen participants completed the baseline questionnaires, however one participant’s circumstances changed immediately after completing the baseline measures so withdrew from the study before randomisation. Therefore, eighteen participants were stratified before being randomly allocated to the intervention (*n* = 9) or control group (*n* = 9). No further contact was established with one of the control participants, leaving 17 participants recruited into the study. Participant enrolment is schematically presented in the CONSORT flow diagram in Fig. 1.

### Feasibility and Acceptability Results

Seventeen participants were recruited over the four-month period. The aim of recruiting 24 participants was therefore not met. All participants consented to be randomised and no participants were lost during the study period. There was minimal missing data (2%); one of the control group participants did not reveal their income band; one control group participant did not complete measures at T1; and one SOLACE group participant missed the courtesy stigma measures (7 items).

Adherence to the intervention was good. One participant withdrew from the intervention after attending one online session but chose to remain part of the study, completing all follow up data, and was therefore included in the analysis. The other participants attended an average of six sessions. The online sessions were better attended than the face to face sessions (t = − 4.01, p < 0.05). Reasons for not attending were lack of childcare, scheduling conflict, or difficulty with bedtime routines.

### Implementation Fidelity

Fidelity to the manual was good. All topics were covered within the time planned. Parents spent less time on the topic of disclosing the diagnosis to others and more time on the topic of if, when and how to disclose the diagnosis to their child. Parents also talked a lot about schooling including issues with teachers and whether or not a special needs educational setting was appropriate for their child.

During the online sessions, a husband of one of the participants joined each session. Because no baseline data was obtained he was not included in the data analysis. However, he did provide consent and was therefore included in the post-intervention focus group interview as he had attended 5 sessions.

Some technical issues interfered with the delivery of the online sessions. During the first session, the sharing of the screen did not work on the first attempt and the sound quality from some of participants’ microphones was poor. To adjust for this, ahead of the second online session, participants were asked to use headsets and a laptop or PC when possible; however, the majority continued to use a tablet or phone.

### Acceptability

#### Focus Groups

The interviews followed a semi-structured topic guide with the aim of considering four key topics: (1) the structure and format; (2) acceptability of intervention content; (3) future suggestions and; (4) outcome measures. All participants were given a pseudonym.

#### Structure and Format

The structure of the sessions was very well received. Participants enjoyed the themes each week and said how they enjoyed the ‘free sharing time’ at the end of each session (*That nice little bit at the end*, Lilly). The group consensus was that a blended format worked in a feasible, acceptable and effective way. Parents reported setting up the Zoom videoconference software on their phone or tablet was straightforward and easy. There were a few technical issues including sound quality (as previously highlighted) and feedback. Parents (n = 2) who used their mobile phone to take part mentioned they did not see the whole group at once but only the person who was talking at the time making conversation less natural. Nevertheless, these technicalities were minor and the overall response from parents was that the positives of the online meetings significantly outweighed such difficulties. The main benefit of the online sessions according to the parents was that they did not have to arrange childcare. Other benefits reported were that it was easier not to have to rush out and being able to participate in the comfort of their own home. One father stated he prefers talking to people online than in-person: *I find it easier online talking to others. I am not very good face to face talking out loud in front of everyone so I like this, I find it a lot easier*.(Mike) All the other parents preferred a combination of the two delivery methods because face to face sessions are easier to establish rapport and trust quickly. *I think what face to face does, that you can’t really do remotely, is that when you meet people directly you build trust that bit more, for this sort of thing being able to open up and sharing of experiences* (Holly).

#### Acceptability of the Intervention Content

None of the participants reported any problems related to acceptability. Being among parents with autistic children seemed to be one of the most important and valued aspects for the parents. Parents mentioned how they liked to receive and share practical support, tips and advice with the group. Participants reported that it was particularly nice to be able to complain or vent about something, knowing that the other group participants would be more likely to understand. *It is nice to talk to people who are in a similar situation, and to be able to have a good complain about things….you do feel very alone with the way your child is behaving and whether you are doing the right thing or whether they are just completely bonkers. Its good to hear other people going through similar things* (Sally)*.*

The other reported benefits of SOLACE related to the actual content of the program. A couple of parents commented that they thought everything was beneficial without further explanations. Other parents mentioned that they learned new things and commented how SOLACE made them learn to be less hard on themselves. Learning about self-compassion and taking more time for self-care were aspects the parents mentioned as especially useful. *I think one good thing about these sessions is it really made me reflect on the things I do quite well with [son] and actually the things I can give myself a bit more of break about because I think I know I am so harsh on myself. ….. And it doesn’t have to be perfect all the time. It is just good enough. And I think that has been the biggest learning point for me. Is that you know, we are alright* (Lilly).

### Suggestions for Future Implementation

All parents felt the sessions ended rather abruptly and responded that they would have liked it to continue for longer. Some parents suggested that weekly sessions may be too much commitment but that monthly on-going sessions beyond the eight weekly sessions would be feasible, beneficial and appreciated. No negative comments or suggestions were made in relation to changing any of SOLACE’s content. There was, however, a suggestion to share the video clips beforehand so that they had more time to process the content. The parents all agreed that they would recommend SOLACE to other parents and carers and mentioned it would be great to see the intervention rolled out more widely.

The opinions about the Facebook group were not particularly strong. They said they found it useful to be reminded about upcoming sessions but few parents were frequent Facebook users so did not engage with the Facebook group much. One mother expressed her concern about visibility on Facebook. Parents suggested that a Whatsapp group may be easier and more useful.

### Outcome Measures

The responses related to discussions about the outcome measures were overall positive. The parents thought there was enough time to complete the questionnaires and that they were not too long. Two mothers mentioned how they found the questions confrontational and one mother reported how completing the questionnaires made her reflect on her well-being in a positive way: *When you first gave me the questionnaire is when I realised I recognised I was feeling pretty rubbish and I think you bury that so far sometimes because I struggle since his diagnosis … because I don’t have a choice, it is my son who I love dearly and you just need get on with it. But it is the last questionnaire made me realise that SOLACE definitely been helpful cause I surely feel better than I did before, even though I didn’t necessarily recognise how rubbish I was feeling, so I think that has been really great (Lilly).*

#### Preliminary Effectiveness

##### Baseline Data

No differences between groups were detected for age, gender, socio economic status, age of children, level of education, religiosity, or use of support groups (*p* > 0.05). All children had received an official diagnosis of autism at the time of completing the baseline questionnaire. Demographic baseline data are summarised in Table 2. Comparisons of all outcome measures at T0, T1 and T2 are displayed in Table 3.

**Table 2:** Participant demographics

	SOLACE	CONTROL	Total*
Gender			
Female (n)	8	8	16
Male (n)	1	–	1
Age			
Range in years	26–42	24–50	24–50
Mean (SD)	35.00 (6.42)	39.25 (8.83)	37.00 (7.71)
Ethnicity			
White British (n)	7	7	14
Black British (n)	–	1	1
Asian (Pakistani) (n)	1	–	1
Maori (n)	1	–	1
Marital status			
Married/Cohabiting (n)	9	7	16
Single(n)	–	1	1
Religion			
None (n)	5	6	11
Christian/Catholic (n)	3	2	5
Islam(n)	1	–	1
Education			
A levels (n)	1	2	3
College (n)	2	1	3
University degree (n)	4	3	7
Masters degree (n)	1	2	3
Doctorate (n)	1	–	1
Employment			
Full time	3	1	4
Part time	3	2	5
Looking for work	–	1	1
Full time carer	3	4	7
Income			
Less than £10.000	1	3	4
£10.000–£19,999	3	–	3
£20.000–£29.999	2	1	3
£30.000–£49.999	1	1	2
£50.000–£79.999	–	2	2
£80.000–£99.999	1	–	1
£100.000 +	1	–	1
Support groups			
Member of (online) support group (n)	6	4	10
Use rarely	1		1
Use not very often	1	2	3
Use sometimes	6		6
Use regularly		2	2
Use often		1	1
Child gender			
Male	7	6	13
Female	2	2	4
Age			
Range in years	3–8	2–10	2–10
Mean (SD)	4.83 (1.73)	7.13 (2.95)	5.91 (2.59)
Diagnosis			
Autism	9	8	17
ADHD	1	1	2
Global developmental delay	2	1	3
Speech and language delay	3		3
Dysphraxia	1		1
Down syndrome		1	1
Dyslexia		2	2

**Table 3:** Means, standard deviations and significant differences between and within group of each outcome measure, at baseline, T1, and T2

	T0		T1		T2	
	Mean (SD)		Mean (SD)		Mean (SD)	
	SOLACE	Control	SOLACE	Control	SOLACE	Control
MHI-5	45.78 (18.88)	49.00 (15.82)	53.78 (21.83)	56.57 (24.81)	60.00 (18.22)^b^	58.50 (19.47)
Courtesy stigma	1.94 (0.61)	2.05 (0.60)	1.25 (0.24)^b^	2.06 (0.50)^a^	1.27 (0.33)^b^	1.84 (0.31)^a^
Affiliate stigma	2.51 (0.48)	2.36 (0.49)	2.09 (0.29)^b^	2.44 (0.42)	2.06 (0.37)^b^	2.29 (0.48)
Self-esteem	13.89 (5.71)	18.13 (5.14)	17.11 (5.30)^b^	16.57 (5.35)	15.56 (5.58)	16.63 (5.50)
Self- compassion	2.91 (0.73)	2.80 (0.80)	2.99 (0.57)	2.43 (0.88)	3.12 (0.62)	2.52 (0.74)
Pos. meaning in caregiving	2.79 (0.43)	2.40 (0.94)	3.15 (0.38)^b^	2.04(1.00)^a^	2.98 (0.43)	2.30 (0.94)
Self-blame	4.06 (0.83)	3.04 (0.84)^a^	3.64 (1.00)	3.44 (1.12)	3.58 (1.22)	3.10 (0.99)
Social support	65.67 (13.11)	49.88 (11.46)^a^	72.00 (13.26)	46.57 (12.41)^a^	68.67 (16.82)	48.06 (13.17)^a^
Social isolation	26.00 (3.35)	26.50 (5.53)	27.33 (3.54)	29.29 (5.28)^b^	20.22 (3.82)^b^	23.88 (3.87)

^a^Significant difference between groups, p < 0.05

^b^Significant change from baseline within groups, p < 0.05 (not Bonferroni corrected)

##### Mental Health

**Fig. 2:**
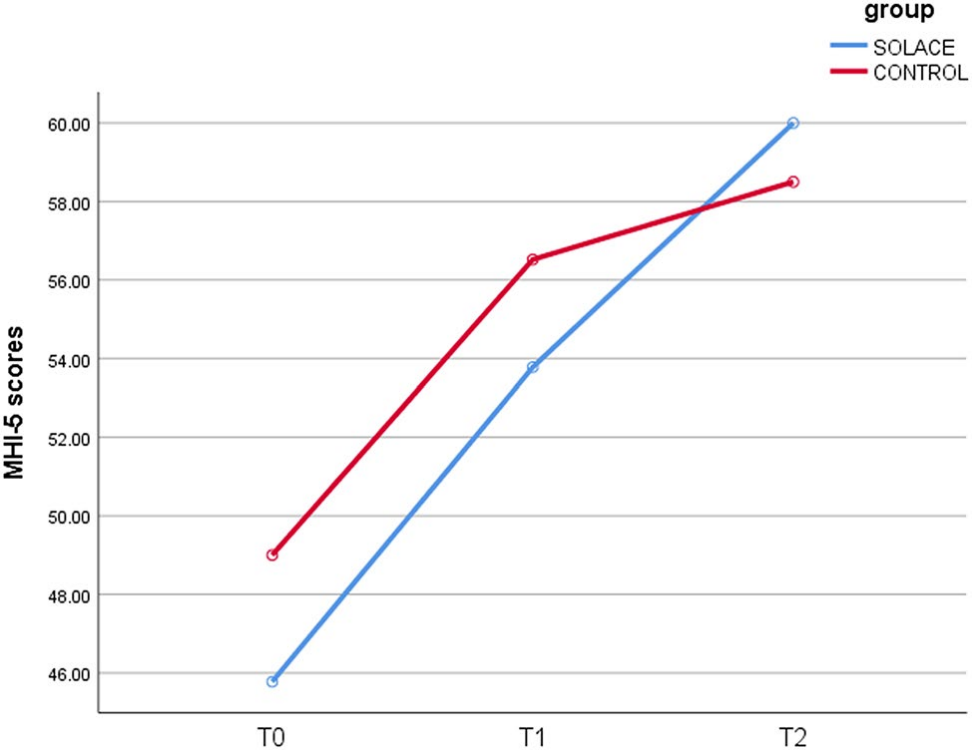
MHI-5 scores over time for both intervention and control group

The mean MHI-5 scores at baseline were 45.78 (SD = 18.88) for the SOLACE group and 49.00 (SD = 15.82) for the control group. At post intervention (T1) the mean scores had improved for both groups with a mean score of 53.78 (SD = 21.83) for SOLACE and 56.57 (SD = 24.81) for the control group. Six weeks after SOLACE finished (T2) the participants from SOLACE reported a higher mean mental health score (60.00, SD = 18.22) than the control group (58.50, SD = 19.47).suggesting a bigger change in mental health scores for those who participated in the SOLACE group. A one-way correlated analysis of variance showed that the positive change in scores for the SOLACE group was significant (Sphericity Assumed) F(2,16) = 4.23, p = 0.034. Further exploration showed that the difference in mental health scores between T0 and T2 was significant: t (8) = 2.49, p = 0.041. For the control group, the differences in mean scores over time were not significant F(2,12) = 3.32, p > 0.05. No significant differences were detected between groups at any time point. Figure 2 illustrates the direction of mental health scores for both groups over time.

##### Secondary Outcomes

Scores of perceived courtesy stigma reduced significantly after for those who took part in SOLACE (t (7) = 2.73, p = 0.03 which remained significant at T2 (t (8) = 2.62, p = 0.03). Between-group analysis found that SOLACE scored significantly lower than the control group at T1 and T2 (t (13) =  − 4.10, p < 0.01 and t (15) = − 3.65, p < 0.05 respectively). For the SOLACE group, affiliate stigma scores also reduced significantly post intervention (t (8) = 3.97, p = 0.004) and T2 (t (8) = 5.90, p < 0.001).

Participants from the SOLACE group reported low self-esteem at the start of the intervention (mean = 13.89 (SD = 5.71). Their scores improved significantly after taking part in SOLACE (t (8) = − 4.59, p = 0.002). However, at the follow up measure point their scores had slightly declined again and were no longer significantly higher than baseline. No significant changes for the control group were observed.

No significant between- or within-group differences were observed in self-compassion scores for either group. Reported positive meaning in caregiving creased significantly for parents in the SOLACE group after the intervention at T1 (t (14) = − 2.82, p = 0.01). This significance was not maintained at follow up. The scores of the control group went in the opposite direction and between group analyses found that SOLACE scored significantly higher in positive meaning of caregiving than the control group at T1 (t (14) = 3.74, p = 0.008).

At the start of the intervention, those in SOLACE reported significantly higher self-blame scores than the control group (t (15) = 2.51, p = 0.008). After the intervention this difference had disappeared and SOLACE’s self-blame scores improved whereas those in the control group declined. This trend continued at T2. No significant changes were observed.

At baseline, those who participated in SOLACE reported significant more perceived social support than those in the control group (t (15) = 2.63, p = 0.02). Although not significant, social support scores increased for the SOLACE group immediately post intervention. The differences between groups were still significant at T1 and T2 (t (14) = 3.91, p = 0.002) and t (15) = 2.79, p = 0.014).

Parents from the control group felt more socially isolated at T1 than those in SOLACE. Nevertheless, parents in SOLACE reported an increase in social isolation scores immediately post intervention but a significant drop in social isolation scores at T2. Compared to baseline scores this difference was significant (t (8) = 4.06, p = 0.01).

Comparisons of all outcome measures at T0, T1 and T2 are displayed in Table 3.

## Discussion

To the best of our knowledge, this study is the first of its kind to evaluate a psychosocial intervention designed to focus on the mental health of parents of autistic children through increasing their resistance to autism related stigma. The high retention rates, minimal missing data, good attendance rate, and positive feedback obtained during the qualitative focus group interview suggest that the intervention and study procedures were acceptable to parents of autistic children. Furthermore, no serious adverse events were reported and despite a few challenges during the online delivery, fidelity to the intervention manual was feasible. The results of the trial also suggest that SOLACE is potentially effective in improving the mental health of parents and carers of autistic children.

Recruitment rates were slower than anticipated and the target sample size of 24 was not achieved. Based on the results from the current study, a future study could widen the recruitment strategy and/or consider widening the inclusion criteria. Participation was limited to those whose children had received a diagnosis in the past year. Future studies could include parents with children diagnosed within the past two years as parents with newly diagnosed children may not be ready to participate in programs focused on the parent instead of their child. Schools and pre-school settings were not approached in the current study due to the timing of recruitment, however, future studies may want to use this as an avenue to recruit more participants. Similarly, a future study may wish to approach diagnostic centres (National Health Service) and general practitioner practices for help with recruitment.

Nevertheless, low recruitment rates are common for feasibility studies, in particular for RCTs. Sully et al. (2013) reviewed a cohort of trials funded by the MRC in the UK and found that between 2002 and 2008, 45% of the trials failed to reach their target sample size. Although recruitment rates were lower than expected, retention rates were particularly high for this population. Sixteen participants completed the questionnaires at T1 (94%) and all seventeen participants who completed the baseline questionnaires completed the follow up questionnaires (100%). Hall and Bierman’s (2015) review of technology-assisted interventions with parents of young children reports retention rates ranging from 50–70% (Bert et al. 2008; Enebrink et al. 2012; Kable et al. 2012; Sanders et al. 2008, 2012; van der Zanden et al. 2012).

The method of delivery is likely to have contributed to the high retention and attendance rate. This is in line with emerging research using videoconference instead of face to face group sessions in the carer population (Banbury et al. 2018; Hall and Bierman, 2015). The value added of videoconferencing is that it is cost-effective and has increased accessibility. Studies suggest that this mode of delivery is particularly well received for people with lower social economic status and those who are most socially isolated (Lipman et al. 2011). There is minimal research available that used videoconference in group settings with parents of autistic children. However, a recent study carried out by Kuhlthau et al. (2019) evaluated the feasibility of an 8-week group intervention to reduce stress in parents of autistic children. They delivered the intervention via videoconference and found that their intervention was acceptable, feasible and potentially effective in reducing stress in parents of autistic children. Although future studies are needed to develop a stronger evidence base, this study is among the first to show that videoconference can be used effectively to deliver a psycho social group intervention.

The online sessions were better attended than the face to face sessions which suggest that despite some of the limitations of video conferencing (e.g. technical issues such as low sound quality) parents found it easier to attend the online sessions. The feedback from the parents was generally very positive and parents stated that the benefits from the online sessions outweighed the negatives. It seemed the participants accepted technical limitations as part of the process. This is in line with previous research where participants did note that IT glitches could be frustrating, but that being part of a group and meeting others outweighed the technical difficulties (Banbury et al. 2018; Damianakis et al. 2016).

Parents reported on average low mental health scores at baseline which is in line with previous research (e.g. Cantwell et al. 2015). An increase in mental health scores was observed for parents from the SOLACE group as well as reduced perceived stigma and self-stigma scores in comparison to the control group. This improvement was maintained at six weeks following the intervention. Parents from both groups reported high levels of courtesy stigma and affiliate stigma (self-stigma) in comparison to previous research (e.g. Chan and Lam 2017; Mak and Kwok 2010; Werner and Shulman 2013). This confirms that stigma is troublesome and pervasive for families with autistic children in the UK. SOLACE was successful in reducing both courtesy and affiliate stigma scores.

Feelings of self-blame were reduced and feelings of positive meaning of caregiving, self-esteem scores and self-compassion increased. The qualitative focus group revealed that learning about self-compassion was something the parents found particularly valuable. Parents also reported the importance of being part of group which is unsurprising as this has been consistently highlighted in previous carer literature (e.g. Broady et al. 2017; Kerr and McIntosh 2000; McCabe 2008). However, the quantitative analysis did not fully support this as social isolation scores increased post intervention. It could be that parents felt concerned about the discontinuation of SOLACE as was highlighted during the focus group interview.

It should also be noted that the participants in SOLACE reported higher perceived social support scores at baseline than the control group despite being stratified based on use of number of support groups participants subscribed to, including online Facebook groups. It was anticipated that this would be an effective proxy for social support, however, given the considerable variation in social support levels between groups at baseline, it can be concluded that our stratifying proxy variable was not suitable. Therefore, a future study that wishes to stratify allocation based on social support levels at baseline may wish to use a valid and established measure of social support instead. Interestingly, the SOLACE group reported on average lower mental health scores at baseline which suggests that higher social support at baseline did not relate to higher mental health scores.

Despite promising results, there are some limitations that are worth noting. While successfully demonstrating feasibility and acceptability, the small sample size of this feasibility trial makes it statistically underpowered and thus statistical inferences, including potential effectiveness, must be viewed with caution. A larger trial would allow the assessments of effectiveness at scale. An additional dedicated study is also needed to confirm the findings hence no Bonferroni adjustments were made due to exploratory nature of this feasibility study (Althouse 2016). Another potential limitation of the study is the reliance on self-report measures. It has been argued that participation in an intervention in itself can lead to better ratings on self-report measures (e.g., Fisher and Katz 2000), leading to potential type II errors.

Finally, the qualitative focus group interview was carried out by the facilitator of the intervention. Good rapport was established between participants and facilitator which was essential for the intervention delivery. However, this may be viewed as a limitation of the qualitative data collection. This is because although participants were reminded that the interviews were confidential and encouraged to share their views whether negative or positive, participants may have felt inhibited to share negative views regarding the intervention. A future study would benefit from independent evaluators.

## Conclusion

Prior to this study, no stigma support intervention has been tested empirically with parents of autistic children. The present study provides preliminary evidence that the SOLACE intervention may be effective with this population and although more evidence is needed, health and social care services seeking to implement a practical and impactful mental health support intervention for parents and carers of autistic children should consider using SOLACE. A stigma protection intervention than can be delivered in a widely accessible way would benefit many families and subsequently their autistic children. The currents study demonstrates that a larger trial is warranted.
